# Long-term response of forest productivity to climate change is mostly driven by change in tree species composition

**DOI:** 10.1038/s41598-018-23763-y

**Published:** 2018-04-04

**Authors:** Xavier Morin, Lorenz Fahse, Hervé Jactel, Michael Scherer-Lorenzen, Raúl García-Valdés, Harald Bugmann

**Affiliations:** 1 CEFE UMR 5175, CNRS – Université de Montpellier – Université Paul-Valéry Montpellier – EPHE, F-34293 Montpellier cedex 5, Montpellier, France; 2 Forest Ecology, Institute of Terrestrial Ecosystems ETH Zürich, CH-8092, Zürich, Switzerland; 3 University of Koblenz-Landau, Institute for Environmental Sciences, D-76829 Landau, Germany; 4 BIOGECO, INRA – Univ. Bordeaux, F-33610 Cestas, France; 5 University of Freiburg, Faculty of Biology – Geobotany, D-79104 Freiburg, Germany; 6 Centre of Ecological Research and Forestry Applications (CREAF), Department of Animal Biology, Plant Biology and Ecology, Autonomous University of Barcelona, Cerdanyola del Vallès, Spain

## Abstract

Climate change affects ecosystem functioning directly through impacts on plant physiology, resulting in changes of global productivity. However, climate change has also an indirect impact on ecosystems, through changes in the composition and diversity of plant communities. The relative importance of these direct and indirect effects has not been evaluated within a same generic approach yet. Here we took advantage of a novel approach for disentangling these two effects in European temperate forests across a large climatic gradient, through a large simulation-based study using a forest succession model. We first showed that if productivity positively correlates with realized tree species richness under a changed climate, indirect effects appear pivotal to understand the magnitude of climate change impacts on forest productivity. We further detailed how warmer and drier conditions may affect the diversity-productivity relationships (DPRs) of temperate forests in the long term, mostly through effects on species recruitment, ultimately enhancing or preventing complementarity in resource use. Furthermore, losing key species reduced the strength of DPRs more severely in environments that are becoming climatically harsher. By disentangling direct and indirect effects of climate change on ecosystem functioning, these findings explain why high-diversity forests are expected to be more resilient to climate change.

## Introduction

**Figure 1:**
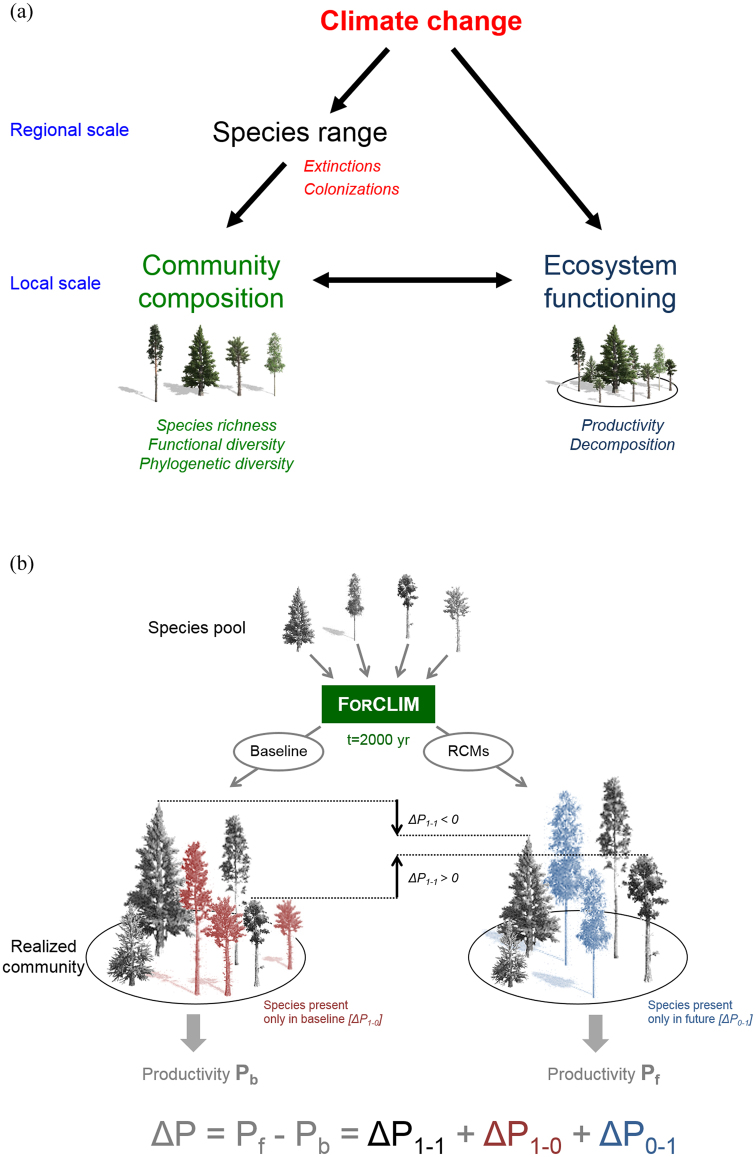
(**a**) Diagram illustrating direct and indirect effects of climate change (including change in CO_2_) on forest ecosystem functioning. (**b**) Schematic representation of the simulation done for one original community composition. ΔP refers to the difference in productivity between anticipated (*i.e*. corresponding to those simulated by one of the RCMs considered here) and baseline climatic conditions. ΔP can be decomposed into three components. ΔP_1−1_ is the difference in productivity between anticipated and baseline climate conditions of species present in both conditions (which may be either positive or negative as represented by the black arrows), ΔP_1*−*0_ decrease in productivity due to species unable to grow in anticipated conditions (red trees in left panel; and ΔP_1−0_ is necessarily negative), and ΔP_0−1_ increase in productivity due to species able to grow only in anticipated conditions (blue trees in right panel; and ΔP_0−1_ is necessarily positive).

Forests are of critical importance globally; they cover ca. 30% of the world’s land surface, harbor most of terrestrial biodiversity^1^, are an important carbon sink^2^, have a pivotal role for climate regulation^3^ and provide many other ecosystem services^4^. Climate change affects forests and their functioning directly (Fig. 1a), including key aspects such as net productivity^2^, via altered abiotic conditions (*e.g*. climate and atmospheric CO_2_ concentration). As a result, a general productivity increase has been observed in forests over the last decades^5^, providing that water was not limiting. However, projections for 2050 suggest that negative impacts of climate change on forest functioning are likely to increase in frequency and intensity, mostly due to severe droughts^6^. Climate change can also affect ecosystem functioning indirectly, through impacts of pests and pathogens^7,8^ or modifications of local communities composition caused by shifts in species distributions^9,10^ (Fig. 1a). Many range shifts of tree species have recently been reported, either in latitude^11^ or elevation^12^, and several examples of local extinctions caused by more severe drought events have been documented, especially at the rear edge of species distributions^13^. Such shifts, which are anticipated to become even stronger in the future^14,15^, lead to changes in local community composition^16^, and possibly affect species interactions^17^. Yet, plant diversity and community composition have been shown to influence ecosystem productivity^18^, although the magnitude of these effects appears to be context-dependent and is not fully understood^19,20^.

For terrestrial ecosystems, diversity-productivity relationships (DPRs) have first been shown experimentally in artificial grasslands^21,22^. The same type of experiments have been set up for forests, but most of them are still young, which limits the relevance of their outcomes^23^. Therefore, DPRs in forests are generally inferred from rather short-term observations using forest inventories^24–26^, showing an overall positive effect of tree diversity on productivity^20^. Yet, empirical studies necessarily include multiple sites that are subject to different environmental conditions, possibly leading to biased results^27^. This is especially true regarding climatic conditions, as climate appears to strongly modulate DPRs in forests^28^. Recently, novel approaches have been proposed to depict and quantify DPRs in tree communities, involving simulations with process-based forest succession models (FSMs)^29,30^. These approaches have the advantage that they can shed light on the mechanisms linking diversity and productivity also in mature forest systems. Furthermore, modelling studies make it possible to test a vast number of combinations of climatic conditions and diversity levels, which is very difficult based on observations due to confounding factors (e.g. mixed forests growing on fertile soils), and practically infeasible in experiments.

While understanding the interaction between climate change and the loss of biological diversity represents a crucial challenge to forecast ecosystem functioning in the future^19,31^, the relative importance of the direct (i.e., through species response to abiotic conditions) and indirect (i.e., through changes in community composition) effects of climate change has not been yet evaluated within the same general approach. As FSMs take both abiotic (climatic, soil) and biotic (competition) factors into account, studies with such models are particularly relevant to disentangle these direct and indirect effects. In other words, owing to such a simulation approach, the changes of productivity can be partitioned into the direct effect of climate change on forest growth *vs*. the indirect effect through modified community composition.

In this study, we therefore used a FSM to quantify the potential relative importance of these direct and indirect effects on forest productivity, and to test how DPRs are theoretically affected by climate change, considering combinations of 30 European tree species and a wide range of environmental conditions in Central Europe (Table S1). To do so, following the approach by Morin *et al*.^29,30^, we simulated virtual forest biodiversity experiments with the FSM ForClim, with various original species richness (1 to 30 European species, with 7,431 original community composition tested) in 11 sites, using either baseline (i.e., current climate) conditions, or future conditions. More specifically, through a large set of simulations, we aimed at:

i. Assessing how climate change affects forest productivity depending on initial environmental conditions;
ii. Quantifying the direct and indirect effects of climate change on forest productivity;
iii. Testing whether the effect of tree diversity (species richness and functional diversity) on forest productivity holds under climate change; and how site-level DPRs would be affected by climate change.

We expect direct effects of climate change to be stronger in the most productive sites, assuming that there is a greater number of species in these sites, with high levels of functional redundancy in terms of functional traits affecting tree growth^32^ that could compensate for decreases or losses of species^29^. In contrast, indirect effects should be prevalent in the less productive sites, where tree species diversity is lower and forest functioning more likely to strongly depend on a few key species^29,33^.

## Results

**Figure 2:**
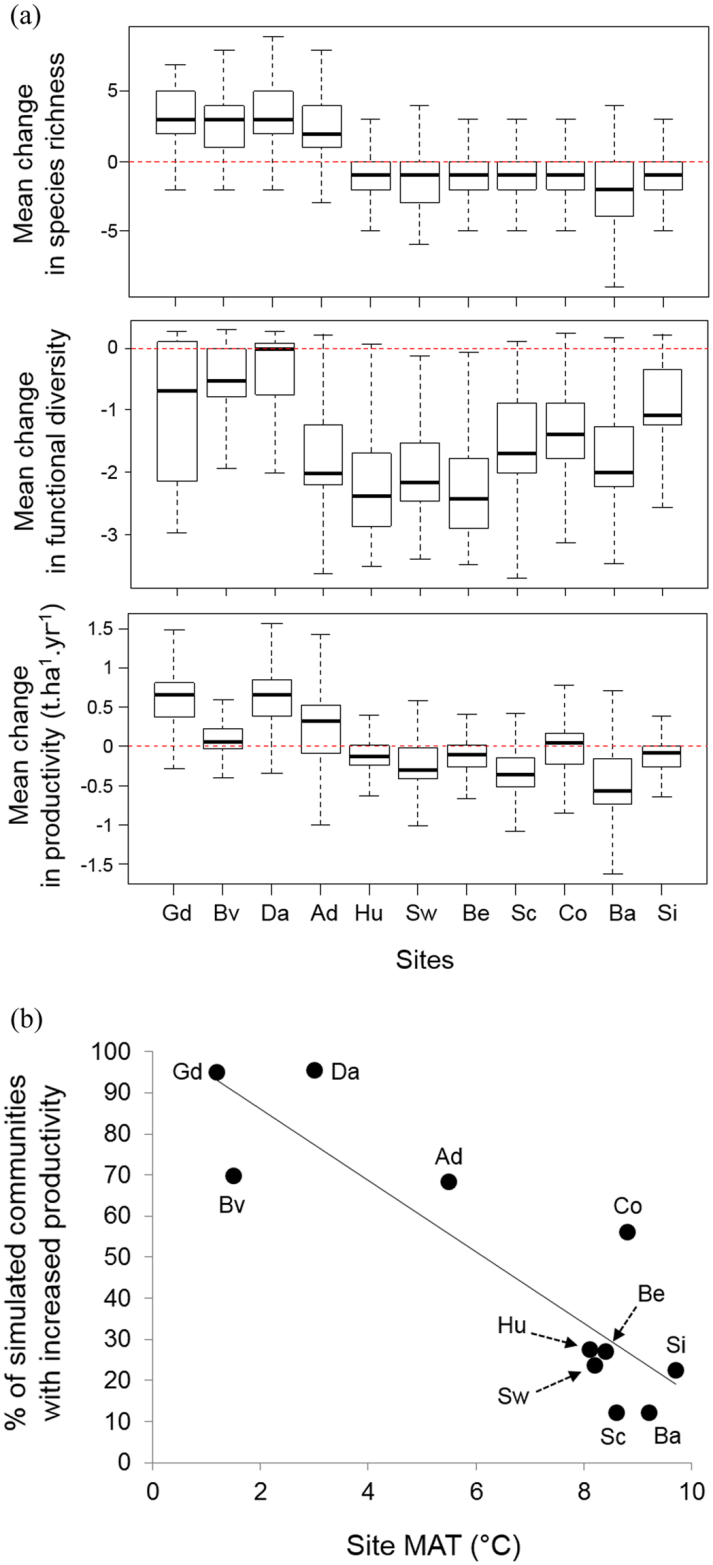
(**a**) Mean changes in species richness, functional diversity, and productivity between anticipated conditions of the KNMI scenario, and baseline climatic conditions, for each site. *n* = 7,431 for each site. Sites are sorted according to their mean annual temperature. *Ad*: Adelboden; *Ba*: Basel; *Be*: Bern; *Bv*: Bever; *Co*: Cottbus; *Da*: Davos; *Gd*: Grande Dixence; *Hu*: Huttwil; *Sc*: Schaffausen; *Sw*: Schwerin; *Si*: Sion. (**b**) Percentage of simulations showing an increase in productivity for each site, between anticipated conditions of the KNMI scenario and baseline climatic conditions against site mean annual temperature (*MAT*) under baseline. *n* = 7,431 for each site. Site names as in (**a**).

The simulated impacts of climate change on forest productivity varied strongly across the gradient of current site conditions, irrespective of their composition. Forests on sites with the coldest conditions experienced, on average, an increase in productivity (hereafter named “*P*+ sites”), while forests on the warmest sites showed a productivity decrease (hereafter “*P−* sites”) (Fig. 2a). For instance, productivity increased by 0.68 (±0.47) t.ha^−1^.yr^−1^ at Davos (site with a mean annual temperature (MAT) of 3.0 °C), but decreased by 0.48 (±0.39) t.ha^−1^.yr^−1^ at Basel (9.2 °C MAT). It appeared that forest productivity was enhanced by the increase in mean temperature in the coldest sites, while it dropped at the other sites because of the decrease in precipitation. However, the interaction between changes in temperature and rainfall could have impacted changes in productivity. For instance, the forests simulated in the site of Bever are on average more productive under future conditions, because this site is the second coldest of the gradient, but this effect is weak because Bever is also one of the driest sites (Fig. 2a). It is also noticeable that the sites with the simulated forests showing the lowest productivity under current conditions were not necessarily *P*+ sites under new conditions (eg. forests simulated in the site of Sion experienced a decreased in their productivity, while they have the weakest productivity under current conditions).

Furthermore, the number of tree species communities predicted to experience improved productivity decreased with higher mean annual temperature (Fig. 2b; Slope = −8.70, r^2^ = 0.77, *P* < 0.001). This finding is consistent with already reported temperature-dependent changes in physiology and productivity^2^.

Differences in climate between baseline and RCM ‘future’ conditions led to different realized forest communities (with different tree species richness) and productivity for the same initial species combination (Fig. 1b). Changes in species richness followed the same pattern as changes in productivity (Fig. 2a), and were positively correlated with each other across all simulations (r = 0.54, n = 81741 (i.e. 11 × 7431), p < 0.001, Pearson correlation). We also tested how climate change affects functional diversity by calculating changes in functional dispersion^34^ between baseline and RCM conditions. A weaker trend than for species richness was found as all sites experienced, on average, a decrease in *FDis* (r = 0.27, n = 81741, p < 0.001). However, the strongest decreases in *FDis* were predicted to occur at *P−* sites, demonstrating that community composition and productivity remain tightly related also in a changed climate (Fig. 2a). To confirm these findings without considering the intra-site variability, we used the median of all simulations at the site level to calculate Spearman correlations across all sites. Changes in richness and changes of productivity were strongly correlated (r = 0.94, P < 0.0001), as well as changes in *FDis* and changes of productivity (r = 0.64, P > 0.0001).

**Figure 3:**
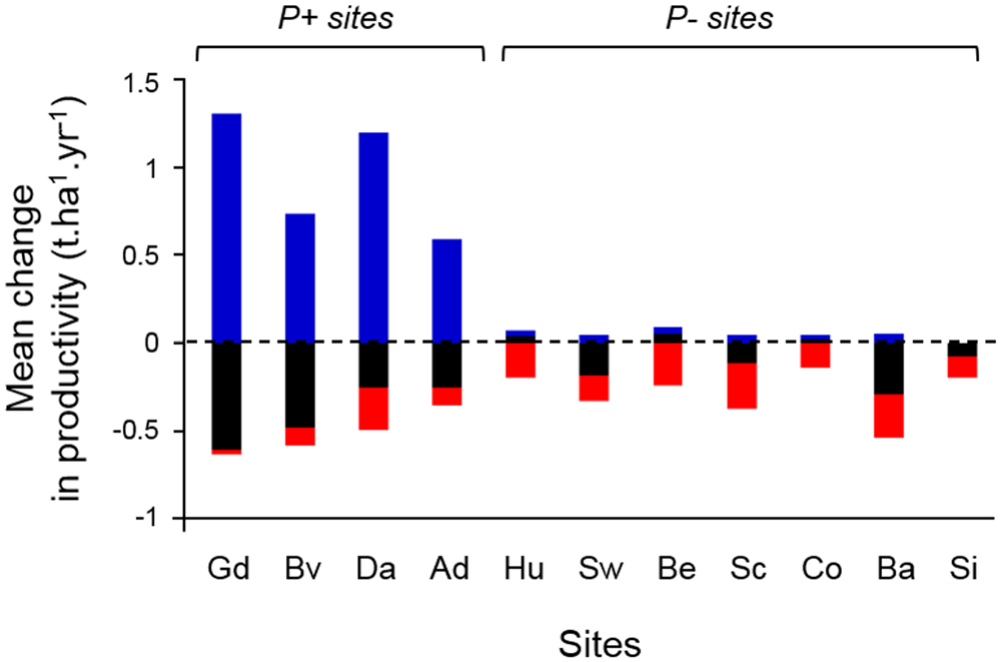
Mean changes in ΔP_1−1_ (black), ΔP_1−0_ (red), and ΔP_0−1_ (blue) (see Fig. 1b legend) across all simulations between anticipated conditions of the KNMI scenario, and baseline climatic conditions, for each site. Sites are sorted according to their mean annual temperature. *n* = 7,431 for each site. Site names as in Fig. 2.

The partitioning of the change in productivity (*ΔP*) between current and future climate showed that the relative importance of *ΔP*_*1*−*1*_, *ΔP*_*1*−*0*_ and *ΔP*_*0*−*1*_ varied strongly across sites. At *P*+ sites, the response of forest productivity to climate change depended primarily on species only able to grow under future conditions (highly positive *ΔP*_*0−1*_, blue bars in Fig. 3), which overcompensated the loss in productivity of species present under both conditions (always negative *ΔP*_*1−1*_, black bars). The response of productivity at *P−* sites was equally determined by local extinctions (negative *ΔP*_*1−0*_, red bars) and by the loss in productivity of species present under both conditions (mostly negative *ΔP*_*1−1*_, black bars). Therefore, at *P*+ sites the productivity increase under climate change was mainly due to warmer conditions allowing for the recruitment and growth of new species, while at *P−* sites communities were affected by local extinctions leading to a decrease in productivity, provided that no colonization by “new” species (i.e. allowed by the new climate conditions) was occurring.

**Figure 4:**
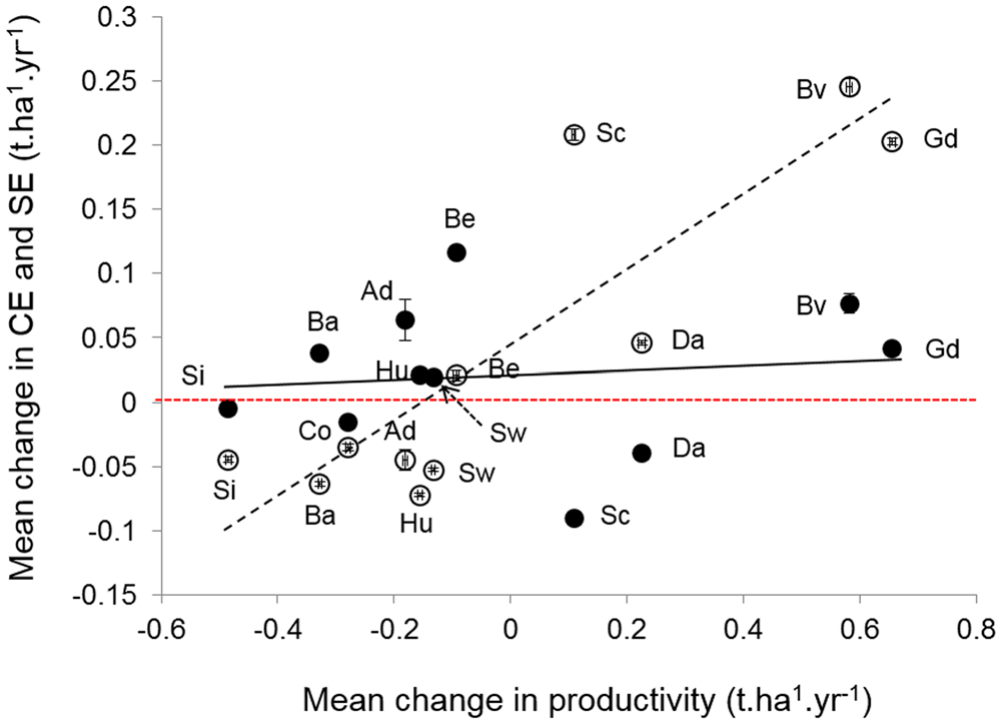
Changes in complementary (open circles) and selection (closed circles) effects *vs*. mean change in productivity between baseline and anticipated RCMs conditions (KNMI). Site names as in Fig. 2.

The partitioning of the net diversity effect showed that changes in productivity between current and future climate were mostly driven by changes in complementarity effects and depended on site conditions (Fig. 4a). At sites where productivity was reduced under climate change (*P−*), we observed weak changes, with a relative increase of selection effects and a relative decrease of complementarity effects. At sites benefitting from climate change (*P*+), the changes in complementarity were strong, especially for 3 sites (Fig. 4a). In these *P*+ sites, *i.e*. those notably receiving “new” species (i.e. species able to grow and survive under RCM conditions but not under baseline conditions), the increase of productivity was thus mainly triggered by stronger complementarity effects (Fig. 4).

**Figure 5:**
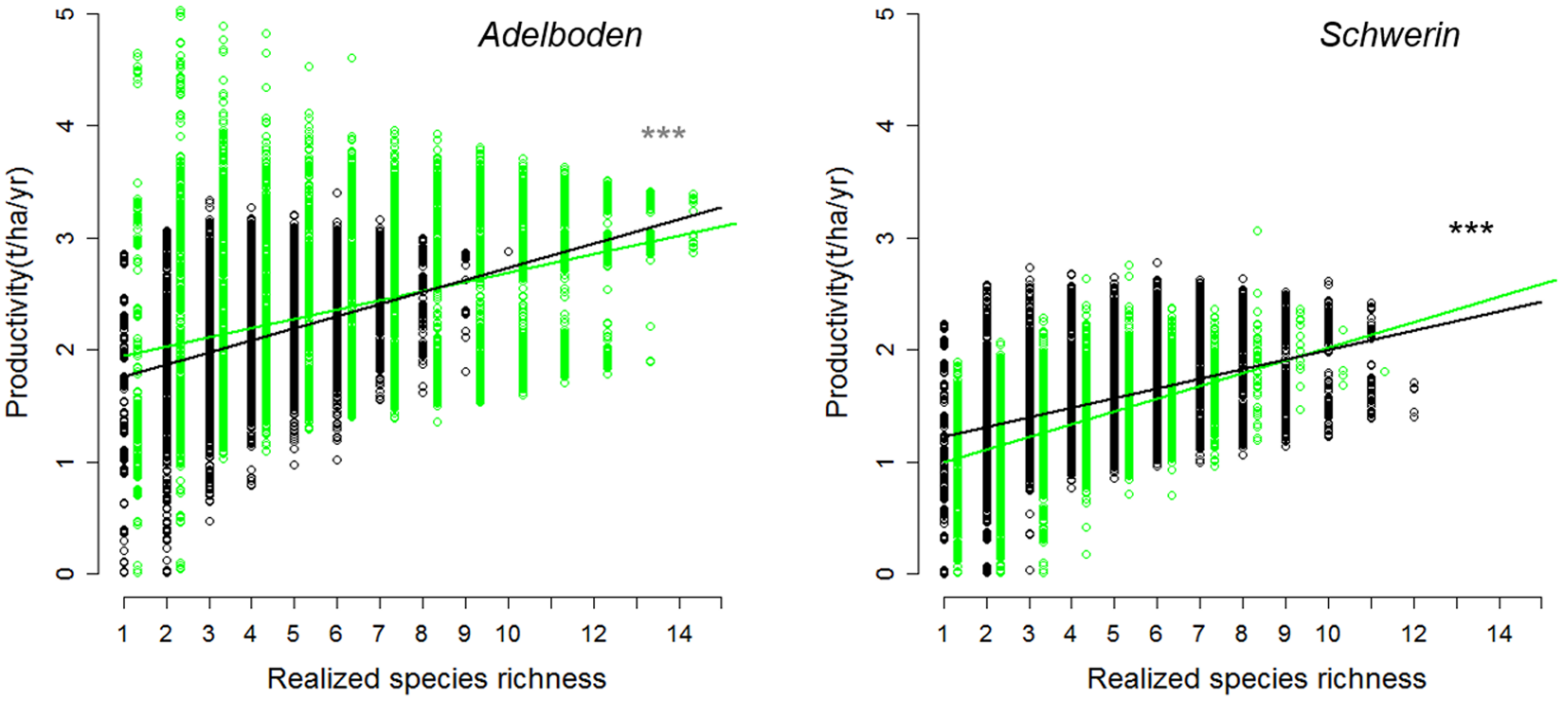
Forest productivity against increasing realized species richness, for baseline (black dots, with regression line in black) and anticipated RCMs conditions (KNMI) (green dots, with regression line in black) for a *P*+ (left panel) and a *P−* site (right panel). Each dot corresponds to one simulation. For each conditions: n = 7,431 simulations. For each site, the slope estimates were significantly different.

We found that forest productivity strongly increased with realized species richness under both baseline and RCMs conditions (see Fig. 5 for the detailed DPR diagram for Adelboden [*P*+ site] and Schwerin [*P−* site]; and Fig. S1 for all sites). The simulations further indicated that DPRs were affected by climate change but only in terms of magnitude, and according to predicted change in site productivity under future conditions. DPRs largely strengthened at *P−* sites, but showed variable responses at *P*+ sites, regardless of the RCM considered (Tables 1 and S4, and Fig. 5). The *P−* sites mostly showed stronger DPRs, with steeper slopes (Table 1 and Fig. S1). Among the 21 DPRs evaluated at *P−* sites (7 sites x 3 RCMs), 15 showed significantly larger slope estimates than the corresponding DPR under baseline conditions; one was significantly weaker and five were non-significant (Tables 1 and S4). Thus, these results showed that under more stressful conditions (*i.e*. in *P−* sites), losing one tree species from the forest community would generally be more detrimental than under current conditions.

**Table 1:**
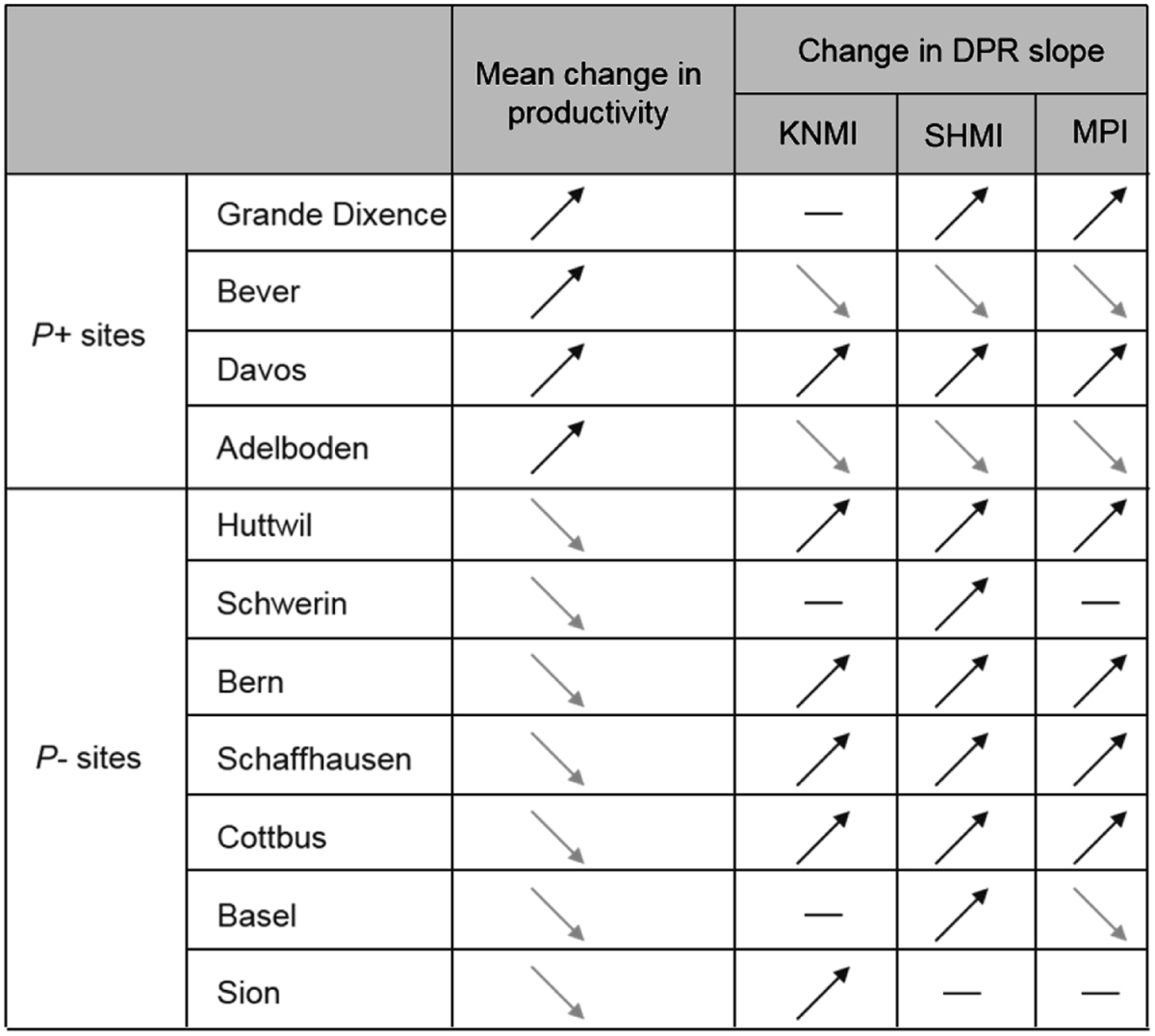
Direction of the mean change in productivity across all simulations, and direction of the change in the slope of diversity-productivity relationships (DPRs) between baseline and anticipated RCMs conditions for all sites and all RCMs (KNMI, SHMI, MPI). *Mean change in productivity:* upward pointing arrow: forest productivity is larger under RCMs conditions than under baseline conditions (i.e. *P*+ site); downward pointing arrow: forest productivity is larger under baseline conditions than under RCMs conditions (i.e. *P−* site). Note that only one arrow was used as the trend was consistent for all RCMs for all sites. *Change in DPR slope:* upward pointing arrow: Slope of DPR under the RCM conditions steeper than under baseline conditions; downward pointing arrow: Slope of DPR under baseline conditions steeper than under the RCM conditions; flat hyphen: no significant difference between the two slopes. Slope values are shown in Table S4, for all RCMs conditions. Mean changes in productivity for each RCMs are shown in Table S3.

## Discussion

Based on an extensive simulation design, our results first confirm that climate change is likely to strongly affect the productivity of temperate European forests^2,5^, but they demonstrate that its impact varies greatly in direction and also in magnitude across sites. Second, our results demonstrate that forest productivity positively correlates with realized tree species richness even under a changed climate, *i.e*. as it is getting warmer and drier, for a large panel of baseline conditions. Third, the outcomes of the simulations reveal that although both direct and indirect effects of climate change significantly affect forest productivity, indirect effects related to changes in community composition are pivotal. On average, indirect effects amounted to 71.3% of the changes in productivity across all simulations (site-level averages ranged from 50.3 to 90.2%). This occurred mostly because new climatic conditions promote the recruitment of new species at the coldest sites, whereas they cause local extinctions at other sites. Overall, this study is the first aiming at quantifying both direct and indirect effects within the same integrated approach, and it emphasizes the importance of considering the role of biodiversity when assessing climate change impacts on ecosystem productivity^9,19^.

The strong effect of climate change on site-level slope of DPRs notably leads to a greater effect of tree species richness on productivity at *P−* sites (*i.e*., at sites expected to experience a decrease in productivity under climate change irrespective of forest composition). Three main causes may interact to generate this effect in our simulations, as discussed below. The first one relates to changes in the strength and direction of species interactions along the lines of the stress gradient hypothesis^35,36^, which predicts that interactions between species will shift from negative (competition) to positive (facilitation) along stress gradients. Extending this hypothesis, we posit that interspecific competitive interactions will decrease in intensity with increasing abiotic stress. Such a change leads to a stronger dependence of ecosystem functioning to species richness in *P−* sites compared to *P*+ sites, because interspecific competition becomes relatively less detrimental for growth in *P−* sites, which is consistent with our results. In fact, at *P−* sites, forest productivity was more reduced by the loss of a tree species under new climate conditions than under baseline conditions, mostly because such a loss led to a relatively larger decrease in complementarity between species. Adding a species to a community should increase interspecific competition for light. Yet, at sites experiencing strong, ecologically detrimental climate change, the increase in competition was, on average, relatively lower than under baseline conditions. This is consistent with National Forest Inventory data showing that overyielding is stronger at low-productivity sites when comparing monospecific with mixed stands^24,37^, as well as with a recent study highlighting that the slope of DPRs was steeper in sites with harsher climatic conditions across a latitudinal gradient of natural forests^28^ – which was also the case in our simulations^29^. However we should acknowledge that no positive interactions (*e.g*. facilitation) are included in the model, although they may be of some importance under dry conditions^38^. Furthermore, our partitioning of the net diversity effect (Fig. 4) confirmed that the decreased diversity experienced by the simulated forests at *P−* sites under future conditions led, on average, to a decrease in the strength of the complementarity effect and to a slight increase in the selection effect. This is probably due to the fact that the reduction of the species pool made these simulated forests more sensitive to the presence of a few key productive species.

Second, our simulations showed that forest communities experienced major changes in their species composition across the entire gradient under climate change (Fig. 2a), which appeared to be the strongest driver of changes in productivity in either *P*+ or *P−* sites. Climate is actually a main determinant of the local species pool through its effect on environmental filtering and biotic interactions^33^. Regarding species interactions, we have already discussed above how the change in the strength of species interactions may interact with the change in species composition driven by new climatic conditions to explain the effect of climate change on DPRs. However, evidence for the role of climate change-induced extinctions and colonizations in forest productivity changes (Fig. 3) is strongly related to the effect of climate on environmental filtering. Therefore, our findings illustrate how changes in tree recruitment could play a key role on forest composition and functioning under climate change^39,40^.

Third, our findings may partially be explained by the change in forest structure along climatic gradients^41–43^, notably an increase or decrease in stand-level basal area. However, as already mentioned, this study focused on long-term dynamics and thus did not consider the transient phase, because our approach to depict DPRs relies on forests at pseudo-equilibrium^29^. Therefore, the simulated pair-wise forests, i.e. with the same original composition for baseline and future conditions, generally show similar structures. However, in the case of strong changes in composition, changes in stand structure can occur and interact with changes in the strength of species interactions.

It is noteworthy that indirect effects mostly arose from the possibility that during a simulation, a species may go extinct or colonize a site because of the effect of climatic conditions on the ability of the species to establish seedlings in this site (regeneration) and on its competitive ability (that depends on tree growth) relative to the co-existing species. Therefore, this study was, to our knowledge, the first to explore the relative weight of direct and indirect effects. Relying on forests at pseudo-equilibrium in terms of biomass, these simulations should obviously not be interpreted as short-term predictions of climate change impact, but rather as an assessment of impacts of changing climate conditions on forest productivity and underlying processes in the long term. The goal of this study was to test how climate change may affect the DPRs without biogeographical implications (e.g. species migration), through a fair and robust comparison between baseline (“current”) and anticipated (“future”) climate conditions. This could only be obtained by using the same simulation design for both situations (i.e. starting with bare ground and with a runtime of 2,000 years).

To complement these findings on long-term dynamics and basic processes, future work should focus on transient dynamics, to include extinction and colonization with greater accuracy, for instance using a spatial-explicit framework at the regional scale^44^, e.g. projections of species distribution models^45^. However, considering changes in community composition also related to species range shifts was not relevant in the present study because doing so would have supposed to precisely focus on transient dynamics at the regional scale, which was impossible to do with sufficient robust confidence with this kind of model^46^ or with certain caution^47^. Furthermore, it would have necessitated a completely different study design, by considering a restricted species pool, different for each site, i.e. including only the species biogeographically present in the region to which each site belongs. Doing so would necessarily affect the between-site comparisons as the simulations would not rely on the same species composition in each site. Finally, this would have required to parameterize the model for other species (like Mediterranean trees and shrubs). However, it is noteworthy that our simulations took into account climate-induced extinctions, although coarsely, as well as colonization by species embedded in the model. Furthermore, regarding the relative strength of direct and indirect effects, coupling our simulations with predictions of species range shifts to also take into account the possible colonization by other species would have necessarily increased the relative importance of indirect effects, and would have thus probably strengthened our main conclusion.

One limitation of our study is that simulations tested the impacts of climate change on community composition mainly through higher temperatures and more severe droughts. However, regarding biotic interactions, the current version of the model focuses on competition for light (although mediated by climatic and soil factors), and did not consider competition for nutrients nor competition for water, which may also be a key process affecting future species assemblages under climate change, particularly under a drier climate^6,48^. For instance, we may expect that the trend depicted here regarding the importance of indirect effects could be amplified by considering competition for water in the driest sites because of a larger number of species disappearing. This study also did not consider the impact of abiotic disturbances in the climate change scenarios because we chose to focus on the dual impact of climate change through the direct and indirect effects. However, incorporating this other kind of impact through increased mortality events may be possible in a modelling study using the same kind of models than here. In addition, our model is not accounting for changes in trophic interactions, such as insect herbivory or pathogen attack, which may also change under future climate^8,49^ and which affect DPR^50,51^.

This study shows how tree species interactions and community composition may change under climate change in the long term, which can in turn strongly affect ecosystem functioning, across a wide range of site conditions. These results represent a baseline for predicting change in mean productivity and DPRs in response to climate change in the long term. It could be supplemented by the inclusion of other processes, like species range shifts^44^ or biotic (e.g. pest outbreaks) or abiotic (e.g. windstorms) disturbances. However, model complexification comes at the cost of lower precision and robustness whereas our model works with only few parameters and is applicable to a large range of species and environmental conditions.

Relying on the emergent properties of the simulations, our results illustrate how new climatic conditions may affect forest productivity in the long term. The strong evidence we found regarding the strength of biotic indirect effects of climate change on ecosystem functioning, through both species loss and recruitment of new species, stresses the key role of biodiversity in promoting ecosystem resistance and resilience to climate change^30^, and thus highlights the need to better understand how species interactions and coexistence processes may shape the link between community composition and ecosystem functioning^33,52^.

## Methods

### Forest succession model

We used ForClim v2.9.6^53,54^, which had been developed for simulating the long-term dynamics of temperate forests over a wide range of environmental conditions. The model is based on a minimum number of ecological assumptions, with few parameter requirements. ForClim follows the standard approach of gap models^46,55^ simulating the establishment, growth, and mortality of trees on multiple forest patches, and deriving forest stand properties by averaging the properties simulated at the patch scale^54^. More precisely, (i) the forest stand is abstracted as a composite of many small patches of land (800 m^2^), each patch having its own dynamics; (ii) patches are horizontally homogeneous, *i.e*. tree position within a patch is not considered; (iii) the leaves of each tree are located in an indefinitely thin layer at the top of the stem; and (iv) successional processes can be described on each of those patches separately, *i.e*. there are no interactions between patches. It considers abiotic and biotic limitations to tree establishment and growth, specifically growing degree-days, soil moisture and nitrogen status as well as light availability at the height of tree crown, *i.e*. outco15mes of inter- and intraspecific competition.

The accuracy of ForClim in Europe has been shown, among others, by its ability to reproduce vegetation patterns and forest biomass along a broad climatic gradient^53,54,56^ spanning 11 sites in central Europe. We have thus focused on these same 11 sites, i.e. sites with very different forest types (representative of central Europe forests) and with mean annual temperature ranging from 1.2 to 9.7 °C, and annual precipitation sums from 573 to 1,350 mm (see Table S1).

Trees get established with a diameter at breast height of 1.27 cm as a function of species-specific responses to winter temperature, light availability at the forest floor, growing degree-days and browsing pressure^53^. Patches being set to be horizontally homogeneous, there is no intra-patch variation in light availability at a given height, and thus at the ground level. The rationale for doing so is that the patch size is usually small (800 m^2^ in this case), representing the area impacted by a single dominant tree. Browsing only affects seedlings in the model. Browsing sensitivity varies across species, and each site has a specific browsing pressure that is kept constant over time. Principally, all species (from the species pool chosen) are available for establishment, *i.e*. there is no dispersal limitation in the model and the trees are supposed to come from surrounding forests.

Growth (i.e., stem diameter increment at breast height) is modeled using an empirical equation derived for optimally growing trees^57^. Actual tree growth is calculated by modifying the optimum rate to the extent that abiotic or biotic conditions are limiting. Specifically, these limiting conditions are defined by growing degree-days, soil moisture and nitrogen status, crown length, as well as light availability at the height of tree crown, i.e. leading to inter- and intraspecific competition and thus changes in species composition. In the version we used, the model concentrates on competition for light. The amount of light available to each tree depends on self-shading as well as shading by taller trees within the patch, thus rendering tree height an important variable. Light availability across canopy is calculated using the Beer–Lambert law for the absorption of light travelling through the leaf layers of each patch, as follows:

1 $$L{A}_{h}={e}^{-k.\sum _{i=1}^{{N}_{h}}LA{I}_{i}}$$

where *LA*_*h*_ is the light availability at height *h* in the canopy, *k* is a coefficient of attenuation (with *k* = 0.25), *LAI*_*i*_ is the leaf area index of the tree *i*, and *N*_*h*_ is the number of trees with a height superior to *h*.

Other resources, such as nitrogen and water, are affecting species performance and vary across sites. While nitrogen availability is a constant at the site level, water availability can vary across years and there is no explicit competition for water between co-existing trees. To calculate weather-dependent factors, mean monthly temperatures and monthly precipitation sums are used. The model is further constrained by soil water holding capacity to calculate a hydric budget for each year in each site. From diameter at breast height, the sizes of other tree compartments (*e.g*. foliage, roots) and total aboveground biomass are estimated using allometric equations  that partly respond to changing competition and thus to diversity changes^53,54^. Species coexistence in forest gap models occurs from two main mechanisms: first, trade-offs evident from life-history strategies, such as high rates of colonization often being tied to low shade tolerance, or typically short lifespans of early successional, fast-growing trees; and second, the fact that cyclical succession is occurring on each individual patch, such that species with different properties are able to dominate during different parts of the cycle. Tree mortality is stochastic and has a background and a growth-related components. The former depends on species maximum longevity, whereas the latter is an integral proxy for stress conditions, i.e. tree vigor; since competition affects individual tree growth, it also has an indirect effect on simulated mortality rates via growth-related mortality^53^. Species parameters are provided in Table S2.

To summarize the role of climate in ForClim, climatic conditions and annual weather variability directly control tree establishment (in addition to other factors), strongly influence tree growth, and have an indirect effect on survival (i.e., via growth). Regarding growth, cold and dry conditions limit individual productivity (with an intensity that depends on species characteristics – see Table S2), whereas trees are close to their optimal growth under moist and warm conditions (obviously without taking into account the effect other factors such as light availability across the canopy). A more detailed description of the model and its development over time can be found in several publications^53,54^. ForClim has evolved from a simulator of forests in the Swiss Alps to a general model that is applicable to temperate forests of central Europe^53,58^, eastern North America^58^, the Pacific Northwest of the US^59^, northeastern China^60^ and the Colorado Front Range of the Rocky Mountains^61^. To our knowledge ForClim is a forest succession model that has been demonstrated to be applicable “out of the box”, *i.e*. without any re-parameterization, across widely different climates while still keeping a species resolution, which supports its generality. Note that using a succession model to explore the diversity-productivity relationship differs from previous modelling studies^62^ because: (i) we used a multi-trait model that takes into account observed trade-offs in species biology (e.g. growth/shade tolerance), as the ForClim parameters are mostly derived from observed/measured traits; and (ii) the model has originally not been developed to study diversity-productivity questions, and can thus be viewed as an independent tool, as illustrated in former studies^29,30^.

### Climate data

To represent the anticipated change in climate in the 11 sites (see Table S1 for a detailed description of the site conditions), climate data for the 21^st^ century were obtained from the Landscape Dynamics Unit at the Swiss Federal Institute for Forest, Snow and Landscape Research (WSL). Data were spatially interpolated at 1 km resolution. Three Regional Climate Models (RCMs) nested in the General Circulation Model ECHAM5 were used to derive climate data based on the IPCC^63^ AR4 SRES scenario A1B, providing us with the alternatives KNMI (*Royal Netherlands Meteorological Institute KNMI RACMO2*), SMHI (*Swedish Meteorological and Hydrological Inst. SMHI RCA30*), and MPI (*Max-Planck-Institute for Meteorology MPI CLM*). To derive anomalies, we used the periods 1950-2000 for KNMI, 1961–2000 for SMHI and 1960–2000 for MPI from the WorldClim dataset^64^ as baseline periods and the period 2090–2100 as representative for future conditions. Averaged changes in mean annual temperature and annual precipitation sums between the two periods are shown in Table S3. Note that we chose to use one SRES scenario but several RCMs because variability was greater between RCMs than between scenarios from IPCC^63^. Similarly to Rasche *et al*.^65^, we averaged the nine cells covering and surrounding each of our 11 locations.

We used simulated time series data with stationary climatic conditions (*i.e*. no trend across time, but including inter-annual variability) for both baseline and anticipated climate, to run the simulations over 2,000 years because the main objective of this study was to explore how climate change affects the DPR from a theoretical point of view, and not to produce robust predictions of changes in DPR in the next century. From the transient RCM data, we thus only used the years 2090–2100, repeating these years randomly over a 2,000-yr period to consider anticipated climatic conditions that were also assumed to be stationary.

### Simulations

#### Virtual diversity experiments

Following the approach presented in Morin *et al*.^29^, we performed simulations with ForClim, with various original species richness (1 to 30 European species) for the 11 sites. For each site, we tested 7,431 original species combinations (containing *n* = 1 to 30 species, with all odd numbers *n* between 3 and 27; see below), under both current (baseline) and future conditions based on output from three Regional Climate Models (RCMs). A total of 326,964 simulations were carried out (4 climate conditions x 11 sites x 7,431 species combinations). Each simulation was run over a time period of 2,000 years, starting from bare ground to avoid any effect due to transient dynamics, and over 200 patches (patch size^1^/_12_ ha, *i.e*. stand of 16 ha in total per simulation) (Fig. 1b). As the results were very similar across RCMs, we illustrate the results mostly based on the KNMI RCM.

To warrant that the simulated forests at the end of the simulation run were at pseudo-equilibrium (*i.e*. a state in which total biomass varies weakly across years, but where gaps continue to occur randomly in the forest), we considered the last 1,000 years of each simulation run^29^. For each simulation, we collected the realized species richness (*i.e*. final richness at the end of the simulation), relative abundance and mean productivity of each species. Mean productivity values were calculated by averaging the yearly productivity (newly accumulated biomass) over the years 1100, 1200, …, 2000 (*i.e*., 10 samples at decadal intervals over 100 years) in order to minimize temporal autocorrelation.

#### Number of simulations with various tree diversity

At each site we ran simulations that differed in their original species composition, ranging from 1 to 30 European tree species for which ForClim had been parameterized (see list of species and parameters in Table S2). However it was not feasible to simulate all possible combinations of species, as this would represent

$${\rm{N}}=\sum _{k=1}^{30}(\begin{array}{c}30\\ k\end{array})\approx 1.07\,\ast \,{10}^{9}\,{\rm{simulations}}\,{\rm{for}}\,{\rm{each}}\,{\rm{site}}{\rm{.}}$$

To reduce the number of simulations, we (i) first chose to limit the simulation runs to 500 for each diversity level i.e., number of species *k*. For k = 30, we ran 1 simulation, *i.e*. with all possible species. For *k* = 1 and *k* = 29 we ran simulations corresponding to the

$$(\begin{array}{c}30\\ 1\end{array})=(\begin{array}{c}30\\ 29\end{array})=30\,{\rm{possible}}\,{\rm{combinations}}\,{\rm{for}}\,{\rm{each}}\,\mathrm{site};$$

and for *k* = 2 and *k* = 28 we ran simulations corresponding to the

$$(\begin{array}{c}30\\ 2\end{array})=(\begin{array}{c}30\\ 28\end{array})=435\,{\rm{possible}}\,{\rm{combinations}}{\rm{.}}$$

Then (ii) for *k* > 2 and *k* < 28, we chose to only consider odd numbers to reduce the number of simulations. Therefore, for richness levels *k* = {3,5, …, 25,27}, as the total number of possible combinations was too large, we ran 500 simulations randomly drawn from all possible combinations of species, respectively. Thus, overall we ran

30 + 435 + 13 × 500 + 435 + 30 + 1 = 7,431 simulations for each site and for one climate dataset, differing in their initial species composition.

### Analyses

First, we tested how climate change affected mean forest productivity through direct and indirect effects by comparing the simulations between baseline and RCM conditions with the same original tree species composition. To do so, we partitioned the change in productivity (*ΔP*) as follows:

2 $${\Delta }P={\Delta }{P}_{1-1}+{\Delta }{P}_{1-0}+{\Delta }{P}_{0-1}$$

where *ΔP*_*c*–*f*_  denotes the difference in productivity between current (c) and future (f) climate scenarios according to the absence (c, f = 0) and presence (c, f = 1) of species. Thus, *ΔP*_*1*−*1*_ is the difference in productivity between future and baseline climate conditions of those species present under both conditions, *ΔP*_*1*−*0*_ is the loss in productivity due to species unable to grow under future conditions (*ΔP*_*1*−*0*_ < 0), and *ΔP*_*0*−*1*_ is the gain in productivity due to species able to colonize the patch and grow under future conditions only (*ΔP*_*0*−*1*_ > 0; Fig. 1b).

Second, to test whether DPRs varied with climate change and site conditions, linear regression between productivity and species richness were used to quantify DPRs (as the slope of the regression is a simple way to assess the strength of the relationship, as typically done)^21,22^, for both baseline and anticipated climates. In these analyses, we considered final species richness, i.e. at the end of the simulations. However, because of climate change, the range of species richness may vary between baseline and future conditions, which may create a bias in the comparison of slopes. Therefore we also performed linear regression between productivity and species richness along the same species richness range (i.e. between n = 1 and the lowest maximum richness reached over all simulations in either baseline or anticipated conditions). The estimates calculated for each slope varied very weakly in comparison to the original calculations, and these additional analyses thus led to the same results. Linear regressions between productivity and functional dispersion were also carried-out at the site level for baseline conditions and all RCM conditions. Normality of the residuals was checked using Q-Q plots. Functional diversity was calculated from all species parameters (considered as traits) using the functional dispersion index^34^ as in Morin *et al*.^29^. Linear regressions between productivity and either realized species richness or functional dispersion were calculated at the site level for baseline conditions and all RCM conditions. Normality of the residuals was checked using Q-Q plots.

Furthermore, the net effects of tree diversity on forest productivity can be explained by two basic classes of mechanisms, referring either to the presence of particular species (selection effects) or to a more efficient use of resources in diverse communities due to niche differentiation or facilitation (complementarity effects)^66^. To test for their relative role, we first quantified the net biodiversity effect (in all simulations with more than one species in the community at the beginning of the simulation) as the difference between the simulated productivity of a multi-species forest and its expected productivity under the null hypothesis of additivity (no diversity effect), based on the simulated productivity of monospecific forests of component species, according to their final relative abundance (in terms of biomass) at the end of the simulation. Then we partitioned the net biodiversity effect into selection (*SE*) and complementarity (*CE*) effects^66^, and further divided *SE* and *CE* by the expected forest productivity based on component monocultures to allow for inter-site comparisons.

### Data availability

The data that support the findings of this study are available from the corresponding author upon reasonable request.

## Electronic supplementary material

Supplementary Information
