# Supplementary material for: Long-term response of forest productivity to climate change is mostly driven by change in tree species composition
Source: Sci Rep. 2018 Apr 4;8:5627. doi: 10.1038/s41598-018-23763-y (PMC5884854; doi:10.1038/s41598-018-23763-y)

**Supplementary Information for**

Long-term response of forest productivity to climate change is mostly driven by change in tree species composition

Xavier Morin, Lorenz Fahse, Hervé Jactel, Michael Scherer-Lorenzen, Raúl García-Valdés, and Harald Bugmann

***Supplementary Tables***

*Table S1*. Description of the sites used in the present study (location, mean annual temperature [*Temperature (°C)*], long-term annual precipitation sum, elevation, and the maximum productivity simulated across all simulations performed in each site.

| **Site name** | **Latitude (°N)** | **Longitude (°E)** | **Temperature (°C)** | **Annual precipitation sum (mm)** | **Elevation (m a.s.l.)** | **Maximum simulated productivity (t/ha/yr)** |
| --- | --- | --- | --- | --- | --- | --- |
| Adelboden | 46.5 | 7.6 | 5.5 | 1351 | 1325 | 3.567 |
| Basel | 47.5 | 7.6 | 9.2 | 784 | 317 | 3.230 |
| Bern | 46.9 | 7.4 | 8.4 | 1006 | 570 | 4.546 |
| BeverS | 46.6 | 9.9 | 1.5 | 841 | 1712 | 2.145 |
| Cottbus | 51.8 | 14.3 | 8.8 | 573 | 76 | 2.110 |
| Davos | 46.8 | 9.8 | 3 | 1007 | 1590 | 2.251 |
| GrandeDixence | 46.1 | 7.4 | 1.2 | 1016 | 2166 | 2.005 |
| Huttwil | 47.1 | 7.9 | 8.1 | 1290 | 638 | 4.907 |
| Schaffhausen | 47.7 | 8.6 | 8.6 | 882 | 400 | 3.415 |
| Schwerin | 53.6 | 11.4 | 8.2 | 625 | 45 | 2.814 |
| Sion | 46.2 | 8.6 | 9.7 | 597 | 542 | 1.113 |

*Table S2*. ForClim parameters for the 30 species.

kName: species name

kType: species type grouping parameter (foliage type)

kS: allometric parameter for relating diameter and height growth

kHMax: maximum tree height (m)

kAMax: maximum tree age (years)

kG: growth rate parameter (cm.years^-1^)

kDDMin: minimal annual degree-day sum (°C.day^-1^)

kWiTN: mimimum winter temperature threshold (°C)

kWiTX: maximum winter temperature tolerated for regeneration (°C)

kDrTol: drought tolerance parameter (0: intolerant / 1: tolerant)

kNTol: nitrogen tolerance parameter (1: needs less N for growth / 5: needs more N for growth)

kBrow: browsing susceptibility (1=less susceptible, ..., 5=more susceptible)

kLy: light requirement of tree saplings (0=low requirement / 1=large requirement)

kLa : shade tolerance of adult trees (1=tolerant, ..., 9=intolerant)

kLQ: leaf litter quality (1=fast, 3=slow decaying)

For more details about ForClim, please see the following references:

- Bugmann, H. *On the Ecology of mountainous forests in a changing climate: A simulation study* PhD Thesis thesis, Eidgenössische Technische Hochschule, (1994).

- Bugmann, H. A simplified forest model to study species composition along climate gradients. *Ecology* **77**, 2055-2074 (1996).

- Bugmann, H. A review of forest gap models. *Climatic Change* **51**, 259-305 (2001).

- Didion, M., Kupferschmid, A. D., Zingg, A., Fahse, L. & Bugmann, H. Gaining local accuracy while not losing generality — extending the range of gap model applications. *Canadian Journal of Forest Research* **39**, 1092-1107 (2009).

| **kName** | **kType** | **kS** | **kHMax** | **kAMax** | **kG** | **kDDMin** | **kWiTN** | **kWiTX** | **kDrTol** | **kNTol** | **kBrow** | **kLy** | **kLa** | **kLQ** | **FG** |
| --- | --- | --- | --- | --- | --- | --- | --- | --- | --- | --- | --- | --- | --- | --- | --- |
| Abies alba | E5 | 73 | 60 | 700 | 117 | 641 | -6 | -3 | 0.23 | 3 | 5 | 0.05 | 1 | 2 | 1 |
| Larix decidua | D2 | 72 | 52 | 850 | 170 | 323 | -11 | -1 | 0.25 | 1 | 3 | 0.4 | 9 | 3 | 4 |
| Picea abies | E5 | 96 | 58 | 930 | 171 | 385 |  | -1 | 0.15 | 2 | 2 | 0.1 | 5 | 3 | 2 |
| Pinus cembra | E5 | 40 | 26 | 1050 | 115 | 323 | -11 | -6 | 0.3 | 1 | 4 | 0.2 | 5 | 3 | 2 |
| Pinus montana | E5 | 46 | 23 | 300 | 138 | 436 |  | -3 | 0.37 | 1 | 3 | 0.4 | 9 | 3 | 2 |
| Pinus sylvestris | E4 | 111 | 45 | 760 | 119 | 610 |  | 1 | 0.37 | 1 | 3 | 0.3 | 9 | 3 | 2 |
| Taxus baccata | E5 | 40 | 22 | 2110 | 47 | 1011 | -5 | 8 | 0.23 | 4 | 5 | 0.075 | 3 | 2 | 2 |
| Acer campestre | D2 | 100 | 23 | 170 | 156 | 1062 |  | 8 | 0.33 | 3 | 4 | 0.1 | 5 | 2 | 2 |
| Acer platanoides | D3 | 108 | 32 | 380 | 142 | 1042 | -17 | 10 | 0.25 | 5 | 4 | 0.025 | 4 | 2 | 2 |
| Acer pseudoplatanus | D3 | 100 | 37 | 550 | 125 | 898 |  | 8 | 0.25 | 4 | 4 | 0.025 | 4 | 2 | 2 |
| Alnus glutinosa | D2 | 111 | 31 | 240 | 250 | 898 | -16 | 11 | 0.08 | 3 | 1 | 0.1 | 5 | 1 | 2 |
| Alnus incana | D2 | 80 | 22 | 150 | 266 | 610 |  | 7 | 0.08 | 3 | 1 | 0.2 | 7 | 1 | 3 |
| Alnus viridis | D2 | 100 | 4 | 100 | 531 | 272 |  | -6 | 0.16 | 3 | 1 | 0.3 | 7 | 1 | 3 |
| Betula pendula | D1 | 108 | 29 | 220 | 278 | 610 |  | 9 | 0.16 | 1 | 1 | 0.3 | 9 | 2 | 4 |
| Carpinus betulus | D3 | 70 | 27 | 220 | 177 | 898 | -9 | 9 | 0.25 | 4 | 2 | 0.075 | 3 | 1 | 2 |
| Castanea sativa | D3 | 85 | 33 | 1510 | 142 | 1237 |  | 10 | 0.33 | 3 | 1 | 0.1 | 5 | 2 | 2 |
| Corylus avellana | D3 | 142 | 10 | 70 | 95 | 898 | -16 | 9 | 0.33 | 2 | 2 | 0.2 | 6 | 1 | 3 |
| Fagus sylvatica | D3 | 64 | 45 | 430 | 191 | 723 | -4 | 9 | 0.25 | 2 | 3 | 0.05 | 1 | 2 | 1 |
| Fraxinus excelsior | D2 | 86 | 42 | 350 | 177 | 980 | -17 | 8 | 0.16 | 5 | 3 | 0.075 | 6 | 1 | 3 |
| Populus nigra | D2 | 120 | 36 | 280 | 285 | 662 |  | 12 | 0.08 | 4 | 2 | 0.1 | 5 | 2 | 2 |
| Populus tremula | D2 | 126 | 30 | 140 | 310 | 610 |  | 9 | 0.25 | 2 | 2 | 0.2 | 7 | 2 | 3 |
| Quercus petraea | D3 | 66 | 45 | 860 | 195 | 785 | -5 | 9 | 0.25 | 2 | 4 | 0.2 | 7 | 2 | 3 |
| Quercus pubescens | D3 | 50 | 25 | 500 | 148 | 1011 |  | 9 | 0.33 | 2 | 4 | 0.3 | 7 | 2 | 3 |
| Quercus robur | D3 | 66 | 52 | 1060 | 195 | 1042 | -17 | 9 | 0.33 | 2 | 4 | 0.3 | 9 | 2 | 4 |
| Salix alba | D1 | 80 | 27 | 170 | 278 | 1062 |  | 12 | 0.08 | 3 | 2 | 0.1 | 5 | 2 | 2 |
| Sorbus aria | D2 | 66 | 22 | 180 | 82 | 898 |  | 12 | 0.33 | 4 | 4 | 0.2 | 7 | 1 | 3 |
| Sorbus aucuparia | D1 | 107 | 19 | 110 | 167 | 498 |  | 7 | 0.33 | 3 | 4 | 0.2 | 7 | 1 | 3 |
| Tilia cordata | D3 | 106 | 30 | 940 | 114 | 1339 | -19 | 8 | 0.33 | 4 | 2 | 0.1 | 5 | 2 | 2 |
| Tilia platyphyllos | D3 | 127 | 39 | 960 | 110 | 1339 |  | 8 | 0.25 | 5 | 2 | 0.075 | 3 | 2 | 2 |
| Ulmus glabra | D3 | 127 | 43 | 480 | 153 | 1062 | -16 | 11 | 0.25 | 5 | 3 | 0.075 | 3 | 1 | 2 |

*Table S3*. Mean changes in climatic variables between anticipated (2090-2100) and baseline (1930-2006) conditions for each site and for all regional climate model employed. *ΔTm:* change in mean annual temperature in Celsius degrees. *ΔPy:* change in yearly precipitation sum in percentage of the average annual precipitation sum calculated in the baseline conditions. *KNMI:* Royal Netherlands Meteorological Institute KNMI RACMO2; *SHMI:* Swedish Meteorological and Hydrological Inst. SMHI RCA30; *MPI:* Max-Planck-Institute for Meteorology MPI CLM. *Total:* averages across all sites.

|  | ΔT_m_ (°C) | | |  | ΔP_y_ (%) | | |
| --- | --- | --- | --- | --- | --- | --- | --- |
|  | KNMI | SHMI | MPI |  | KNMI | SHMI | MPI |
| Adelboden | +3.8 | +4.6 | +4.9 |  | -3 | -14 | -3 |
| Basel | +3.9 | +4.7 | +4.9 |  | - | -10 | -10 |
| Bern | +3.8 | +4.5 | +4.6 |  | - | -15 | -10 |
| Bever | +3.8 | +4.6 | +4.8 |  | +4 | -13 | -13 |
| Cottbus | +3.5 | +4.1 | +4.2 |  | +9 | -12 | -14 |
| Davos | +3.6 | +4.1 | +4.2 |  | +8 | -14 | -15 |
| GrandeDixence | +3.4 | +3.8 | +4.0 |  | +13 | -6 | -6 |
| Huttwil | +3.4 | +3.8 | +4.1 |  | +7 | -10 | -13 |
| Schaffhausen | +2.8 | +3.1 | +3.2 |  | +7 | +1 | +1 |
| Schwerin | +3.1 | +3.2 | +3.3 |  | -1 | +3 | -2 |
| Sion | +3.8 | +4.6 | +4.6 |  | +2 | -13 | -13 |
| **Total** | **+3.5** | **+4.1** | **+4.2** |  | **+4** | **-10** | **-9** |

*Table S4*. Slope estimates of the regression of forest productivity against increasing realized species richness, for baseline and anticipated RCMs conditions. *Est.*: estimate; *S.E.*: standard error. Site names in grey: fertilized sites under climate change; sites names in black: non-fertilized sites. Estimate values for anticipated conditions are in black whether larger than under baseline conditions, and in grey whether weaker than under baseline conditions; and bald values correspond to estimates significantly different than under baseline conditions, from a slope comparison test (Howell 2010).

|  | Baseline | |  | KNMI | | SHMI | | MPI | |
| --- | --- | --- | --- | --- | --- | --- | --- | --- | --- |
|  | Est. | *S.E.* |  | Est. | *S.E.* | Est. | *S.E.* | Est. | *S.E.* |
| Adelboden | 0.11 | *0.003* |  | **0.08** | *0.002* | **0.08** | *0.002* | **0.08** | *0.002* |
| Basel | 0.08 | *0.002* |  | 0.08 | *0.003* | **0.09** | *0.003* | **0.04** | *0.003* |
| Bern | 0.07 | *0.002* |  | **0.09** | *0.002* | **0.10** | *0.002* | **0.09** | *0.002* |
| Bever | 0.07 | *0.004* |  | **0.04** | *0.001* | **0.05** | *0.002* | **0.04** | *0.002* |
| Cottbus | 0.06 | *0.004* |  | **0.07** | *0.002* | **0.19** | *0.002* | **0.10** | *0.002* |
| Davos | 0.06 | *0.005* |  | **0.09** | *0.002* | **0.09** | *0.002* | 0.10 | *0.002* |
| GrandeDixence | 0.10 | *0.006* |  | 0.10 | *0.002* | **0.12** | *0.003* | **0.11** | *0.003* |
| Huttwil | 0.07 | *0.002* |  | **0.09** | *0.002* | **0.12** | *0.003* | **0.10** | *0.003* |
| Schaffhausen | 0.08 | *0.002* |  | **0.09** | *0.002* | **0.12** | *0.002* | **0.12** | *0.002* |
| Schwerin | 0.08 | *0.00* |  | **0.11** | *0.002* | **0.10** | *0.002* | 0.08 | *0.002* |
| Sion | 0.02 | *0.001* |  | **0.41** | *0.01* | 0.01 | *0.030* | 0.02 | *0.005* |

Howell, D. C. (2010). Statistical methods for psychology (7th ed.). Wadsworth, Cengage Learning.

***Supplementary Figures***

*Figure S1*. Forest productivity against increasing realized species richness, for baseline and anticipated RCMs conditions (KNMI). Regression lines (dashed lines) and its 95% confidence interval are shown for each site and each conditions (baseline: green; KNMI: yellow). For each condition: n = 7,431. The first row shows the *P+* sites (names in blue), the second and third one shows the *P-* sites (names in red). ***: slope-difference estimate significantly different than 0 (black: greater than 0; grey: lower than 0); ns: slope-difference estimate non-significantly different than 0 (Slope values in Table S4, for all RCMs conditions).


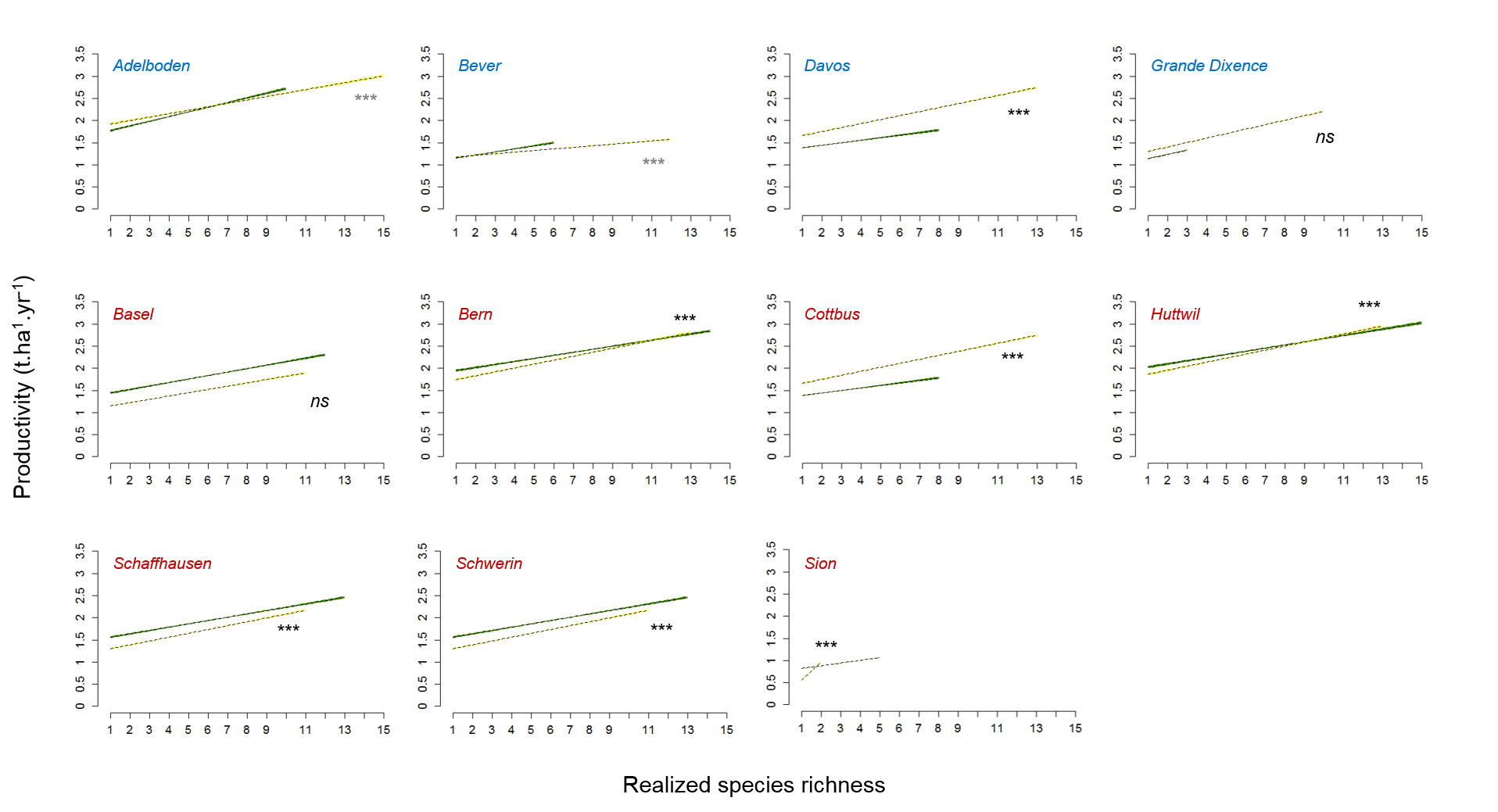

Supplement: Supplementary file 1 — Supplementary Information [file 41598_2018_23763_MOESM1_ESM.docx]
